# Global terrestrial Human Footprint maps for 1993 and 2009

**DOI:** 10.1038/sdata.2016.67

**Published:** 2016-08-23

**Authors:** Oscar Venter, Eric W. Sanderson, Ainhoa Magrach, James R. Allan, Jutta Beher, Kendall R. Jones, Hugh P. Possingham, William F. Laurance, Peter Wood, Balázs M. Fekete, Marc A. Levy, James E.M. Watson

**Affiliations:** 1 Ecosystem Science and Management, University of Northern British Columbia, Prince George, British Columbia, Canada V2N 4Z9; 2 Centre of Excellence for Environmental Decisions and the School of Biological Sciences, The University of Queensland, St Lucia, Queensland 4072, Australia; 3 Centre for Tropical Environmental and Sustainability Science, and College of Science and Engineering, James Cook University, Cairns, Queensland 4878, Australia; 4 Wildlife Conservation Society, Global Conservation Program, Bronx, New York 10460, USA; 5 Ecosystem Management, ETH Zurich, Zuerich 8092, Switzerland; 6 Doñana Biological Station (EBD-CSIC), Avd. Américo Vespucio s/n, Isla de la Cartuja, Sevilla 41092, Spain; 7 School of Geography, Planning and Environmental Management, University of Queensland, St Lucia, Queensland 4072, Australia; 8 Imperial College London, Department of Life Sciences, Silwood Park, Ascot SL5 7PY, Berkshire, UK; 9 Department of Civil Engineering, The City College of New York, New York 10007, USA; 10 Center for International Earth Science Information Network, Columbia University, Palisades, New York 10964, USA

**Keywords:** Conservation biology, Environmental chemistry

## Abstract

Remotely-sensed and bottom-up survey information were compiled on eight variables measuring the direct and indirect human pressures on the environment globally in 1993 and 2009. This represents not only the most current information of its type, but also the first temporally-consistent set of Human Footprint maps. Data on human pressures were acquired or developed for: 1) built environments, 2) population density, 3) electric infrastructure, 4) crop lands, 5) pasture lands, 6) roads, 7) railways, and 8) navigable waterways. Pressures were then overlaid to create the standardized Human Footprint maps for all non-Antarctic land areas. A validation analysis using scored pressures from 3114×1 km^2^ random sample plots revealed strong agreement with the Human Footprint maps. We anticipate that the Human Footprint maps will find a range of uses as proxies for human disturbance of natural systems. The updated maps should provide an increased understanding of the human pressures that drive macro-ecological patterns, as well as for tracking environmental change and informing conservation science and application.

## Background & Summary

Human pressures on the environment are the actions taken by humans with the potential to harm nature^1,2^. Cumulative pressure mapping measures the breadth of these pressures by coupling top-down remote sensing of land cover change with data on additional human pressures collected ‘bottom-up’ through systematic surveys and modelling^3,4^. The method circumvents the limitations of using remote sensing alone, which has difficulty in detecting low intensity pressures^5^, such as linear infrastructures^6^ and pasture lands^7^, and often confounds natural and anthropogenic land covers in arid and mosaic environments^8^.

Cumulative pressure maps have been developed at regional^9,10^ and global scales^11,12^. The ‘Human Footprint’ was first released in 2002 using data primarily from the early 1990s (approximately 1993) on eight human pressures globally, making it the most complete, highest resolution and globally-consistent terrestrial dataset on cumulative human pressures on the environment^13^. It has been used in a large number of ecological and conservation analyses, and still receives around 100 citations each year, particularly from its data users. However, the Human Footprint is a static and dated view of human pressures on the environment. With many of Earth’s systems experiencing pressures close to or beyond safe levels^14^, there is a strong need for an up-to-date understanding of the spatial and temporal trends in human pressures.

Here we use the Human Footprint methodology^13^ to compile remotely-sensed and bottom-up survey information on eight variables measuring the direct and indirect human pressures on the environment in 1993 and 2009. This synthesis represents not only the most current information of its type, but also the first temporally-consistent set of Human Footprint maps, allowing for analyses of change over time. We also provide the first validation of a cumulative pressure map by adopting methods from remote sensing^15^ to visually interpret human pressures in high resolution (median=0.5 m) imagery from 3114 1 km^2^ random sample plots globally (Supplementary Appendix 1). We then determine the level of agreement between these visually interpreted pressures and those mapped by the Human Footprint.

The Human footprint maps provide information on where humans are exerting pressure on natural systems, altering them from their natural states. They also provide information on where these pressures are absent, and ecosystems are likely to be operating in a more natural state. These pressure-free lands represent candidate sites for consideration as ‘Wilderness’^16,17^. The new Human Footprint maps have already been used to show that recent economic and population growth has far outstripped increases in the Human Footprint, yet the most biologically diverse regions of Earth have been disproportionately impacted^18^. We anticipate that the 1993 and 2009 Human Footprint maps will find a range of additional uses, such as serving as proxies for human disturbance and wilderness, including understanding the role of human pressures in driving macro-ecological patterns^19,20^, species extinction risk and distribution analyses^21^, dispersal ecology^22^, conservation science and decision making^23^, and tracking progress toward policy commitments to conservation^23^, among others.

## Methods

### Overview of methods for mapping the Human Footprint

**Figure 1:**
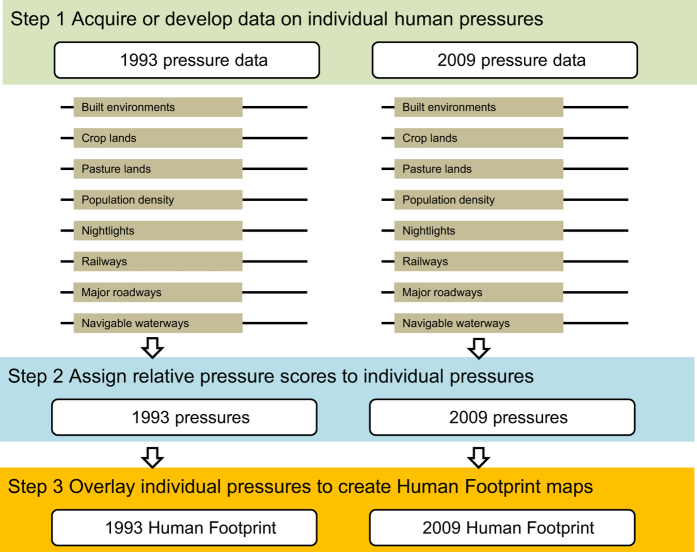
Workflow of the Human Footprint approach to mapping cumulative human pressures on the environment.

To create the Human Footprint maps we adopted the methods developed by Sanderson and colleagues^13^. Data on human pressures in 1993 and 2009 were collected or developed for: 1) the extent of built environments, 2) population density, 3) electric infrastructure, 4) crop lands, 5) pasture lands, 6) roads, 7) railways, and 8) navigable waterways, which are described in detail below (Fig. 1, step 1). To facilitate comparison across pressures we placed each human pressure within a 0–10 scale (Fig. 1, step 2), weighted within that range according to estimates of their relative levels of human pressure following Sanderson *et al.*^13^. The resulting standardized pressures were then summed together to create the standardized Human Footprint maps for all non-Antarctic land areas (Fig. 1, step 3). Pressures are not intended to be mutually exclusive, and many will co-occur in the same location. Three pressures only had data from a single time period, and these are treated as static in the Human Footprint maps.

We used ArcGIS 10.1 to integrate spatial data on human pressures. Analyses were conducted in Mollowedie equal area projection at the 1 km^2^ resolution, yielding ~134.1 million pixels for Earth’s non-Antarctic terrestrial surface. For any grid cell, the Human Footprint can range between 0–50. The following sections and Table 1 (available online only) describe in detail the source data for each pressure, the processing steps applied, and the rationale behind the pressure weighting, and the output datasets created.

### Built environments

Built environments are human produced areas that provide the setting for human activity. In the context of the human footprint, we take these areas to be primarily urban settings, including buildings, paved land and urban parks. Built environments do not provide viable habitats for many species of conservation concern, nor do they provide high levels of ecosystem services^24–27^. As such, built environments were assigned a pressure score of 10.

To map built environments, we used the Defence Meteorological Satellite Program Operational Line Scanner (DMSP-OLS) composite images which gives the annual average brightness of 30 arc second (~1 km at the equator) pixels in units of digital numbers (DN)^28^. These data are provided for each year from 1992 to 2012. We extracted data for the years 1994 (1993 was excluded due to anomalies in the data), and 2009, and both datasets were then inter-calibrated to facilitate comparison^29^. Using the DMSP-OLS datasets, we considered pixels to be ‘built’ if they exhibited a calibrated DN greater than 20. We selected this threshold based on a global analyses of the implications of a range of thresholds for mapped extent of cities^30^, and visual validation against Landsat imagery for 10 cities spread globally.

The DMSP-OLS has limitations for the purpose of mapping human settlements, including hyper sensitivity of the sensors causing detection of over-glow adjacent to built environments^30^ and bright lights associated with gas flaring from oil production facilities^29^. However, no other data exist to map built environments in a consistent way globally over our time horizon. While other datasets provide a one year snap shot of urban extent, they cannot be compared across time due to large differences in the methodologies used^31–33^, and the wildly contrasting extents in mapped built environments.

### Population density

Many of the pressures humans impose on the environment are proximate to their location, such as human disturbance, hunting and the persecution of non-desired species^34^. Moreover, even low-density human populations with limited technology and infrastructure developments can have significant impacts on biodiversity, as evidenced by the widespread loss of various taxa, particularly mega fauna, following human colonization of previously unpopulated areas^35,36^.

Human population density was mapped using the Gridded Population of the World dataset developed by the Centre for International Earth Science Information Network (CIESEN)^37^. The dataset provides a ~4 km×~4 km gridded summary of population census data for the years 1990 and 2010, which we downscaled using bilinear sampling in ArcGIS 10.1 to match the 1 km^2^ resolution of the other datasets. For all locations with more than 1000 people·km^−2^, we assigned a pressure score of 10 (Table 2). For more sparsely populated areas with densities lower than 1000 people·km^−2^, we logarithmically scaled the pressure score using,

(1)Pressurescore=3.333×log(populationdensity+1)

Human population density is scored in this way under the assumption that the pressures people induce on their local natural systems increase logarithmically with increasing population density, and saturate at a level of 1000 people per km^2^.

### Night-time lights

The high sensitivity of the DMSP-OLS^28^ dataset provides a means for mapping the sparser electric infrastructure typical of more rural and suburban areas. In 2009, 79% of the lights registered in the DMSP-OLS dataset had a Digital Number less than 20, and are therefore not included in our ‘built environments’ layers. However, these lower DN values are often important human infrastructures, such as rural housing or working landscapes, with associated pressures on natural environments.

To include these pressures, we used the inter-calibrated DMSP-OLS layers^28^ used for the built environments mapping. The equations for intercalibrating across years are second order quadratics trained using data from Sicily, which was chosen as it had negligible infrastructure change over this period and where DN average roughly 14 (ref. 28). For our purposes, DN values of six or less where excluded from consideration prior to calibration of data, as the shape of the quadratic function leads to severe distortion of very low DN values. The inter-calibrated DN data from 1994 were then rescaled using an equal quintile approach into a 0–10 scale (Table 2). To scale the data, we divided the calibrated night light data into 10 equal sample bins (each bin with a DN greater than 1 contains the same number of pixels) based on the DN values and then assigned them scores of 1 through 10, starting with the lowest DN bin. DN values of 0 were assigned a score of 0. The thresholds used to bin the 1994 data where then used to convert the 2009 data into a comparable 0–10 scale.

### Crop and pasture lands

Crop lands vary in their structure from intensely managed monocultures receiving high inputs of pesticides and fertilizers, to mosaic agricultures such as slash and burn methods that can support intermediate levels of natural values^38,39^. For the purposes of the human footprint, we focused only on intensive agriculture because of its greater direct pressure on the environment, as well as to circumvent the shortcomings of using remotely sensed data to map mosaic agriculture globally, namely the tendency to confound agriculture mosaics with natural woodland and savannah ecosystems^8^.

Spatial data on remotely sensed agriculture extent in 1992 were extracted from the UMD Land Cover Classification^40^, and for 2009 from GlobCover^41^. Although intensive agriculture often results in whole-scale ecosystem conversion, we gave it a pressure score of 7 (Table 2), which is lower than built environments because of their less impervious cover.

Pasture lands cover 22% of the Earth’s land base or almost twice that of agricultural crops^42^, making them the most extensive direct human pressure on the environment. Land grazed by domesticated herbivores is often degraded through a combination of fencing, intensive browsing, soil compaction, invasive grasses and other species, and altered fire regimes^43^. We mapped grazing lands for the year 2000 using a spatial dataset that combines agricultural census data with satellite derived land cover to map pasture extent^42^. We assigned pasture a pressure score of 4, which was then scaled from 0–4 using the percent pasture for each 1 km^2^ pixel.

### Roads and railways

As one of humanity’s most prolific linear infrastructures, roads are an important direct driver of habitat conversion^44^. Beyond simply reducing the extent of suitable habitat, roads can act as population sinks for many species through traffic induced mortality^45^. Roads also fragment otherwise contiguous blocks of habitat, and create edge effects such as reduced humidity^6^ and increased fire frequency that reach well beyond the roads immediate footprint^46^. Finally, roads provide conduits for humans to access nature, bringing hunters and nature users into otherwise wilderness locations^47^.

We acquired data on the distribution of roads from gROADS^48^, and excluded all trails and private roads, which were inconsistently mapped, with only a subset of countries mapping their linear infrastructure to this resolution. The dataset is the most comprehensive publicly available database on roads, which compiles nationally mapped road data spanning the period 1980–2000 and has a spatial accuracy of around 500 m. The gROADS data do not include all minor roads, and therefore should be viewed as a map of the major roadways. We mapped the direct and indirect influence of roads by assigning an pressure score of 8 for 0.5 km out for either side of roads, and access pressures were awarded a score of 4 at 0.5 km and decaying exponentially out to 15 km either side of the road (Table 2).

While railways are an important component of our global transport system, their pressure on the environment differs in nature from that of our road networks. By modifying a linear swath of habitat, railways exert direct pressure where they are constructed, similar to roads. However, as passengers seldom disembark from trains in places other than rail stations, railways do not provide a means of accessing the natural environments along their borders. To map railways we used the same dataset as was used in the original footprint^31^, as no update of this dataset or alternate source has been developed. The direct pressure of railways where assigned a pressure score of 8 for a distance of 0.5 km on either side of the railway.

### Navigable waterways

Like roads, coastlines and navigable rivers act as conduits for people to access nature. While all coastlines are theoretically navigable, for the purposes of the human footprint we only considered coasts^31^ as navigable for 80 km either direction of signs of a human settlement, which were mapped as a night lights signal with a DN^28^ greater than 6 within 4 km of the coast. We chose 80 km as an approximation of the distance a vessel can travel and return during daylight hours. As new settlements can arise to make new sections of coast navigable, coastal layers were generated for the years 1994 and 2009.

Large lakes can act essentially as inland seas, with their coasts frequently plied by trade and harvest vessels. Based on their size and visually identified shipping traffic and shore side settlements, we treated the great lakes of North America, Lake Nicaragua, Lake Titicaca in South America, Lakes Onega and Peipus in Russia, Lakes Balkash and Issyk Kul in Kazakhstan, and Lakes Victoria, Tanganyika and Malawi in Africa as we did navigable marine coasts.

Rivers were considered as navigable if their depth was greater than 2 m and there were signs of nighttime lights (DN>=6) within 4km of their banks, or if contiguous with a navigable coast or large inland lake, and then for a distance of 80 km or until stream depth is likely to prevent boat traffic (Table 2). To map rivers and their depth we used the hydrosheds (hydrological data and maps based on shuttle elevation derivatives at multiple scales)^49^ dataset on stream discharge, and the following formulae from^50,51^:

(2)Streamwidth=8.1×(discharge[m3/s])0.58

and

(3)velocity=4.0×(discharge[m3/s])0.6/(width[m]).

and

(4)Cross−sectionalarea=discharge/velocity

and

(5)depth=1.5×area/width

Assuming second order parabola as channel shape.

Navigable rivers layers were created for the years 1994 and 2009, and combined with the navigable coasts and inland seas layers to create the final navigable waterways layers. The access pressure from navigable water bodies were awarded a score of 4 adjacent to the water body, decaying exponentially out to 15 km.

## Data Records

**Figure 2:**
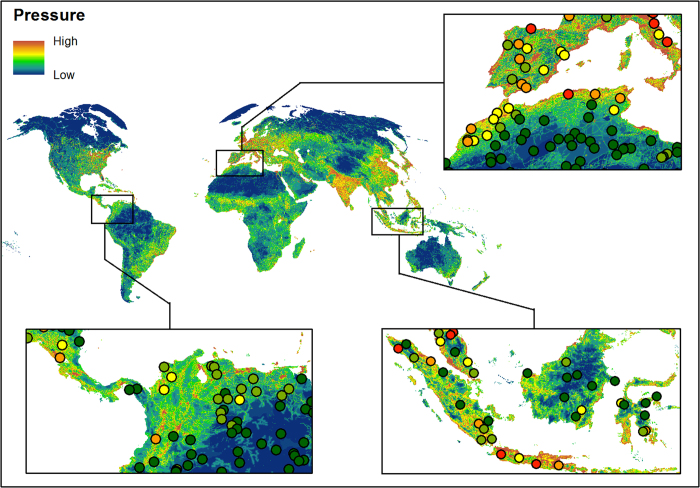
The Human Footprint map for 2009, with panels showing regional overlays with the results of the validation plots.

The 1 km^2^ resolution, temporally-comparable Human Footprint maps [Data Citation 1] are stored in the Dryad Digital Repository, and may also be freely accessed through the Socioeconomic Data and Applications Center website (www.worldpop.org/data/). From Dryad the files may be downloaded as a single 7-zip file archive (7-Zip.org) which contains individual GeoTIFF datasets, an excel file with the validation data and a PDF with the validation key. The GeoTIFFs include the Human Footprint maps for 1993 and 2009 (Fig. 2), as well 14 additional GeoTIFFs of the processed data for each of the eight pressures (Fig. 1, step 2) from each time step (Tables 1,3 (available online only)). The individual pressure layers are provided should data users wish to rework these data to create alternate maps of human pressure for their particular needs or region.

## Technical Validation

**Figure 3:**
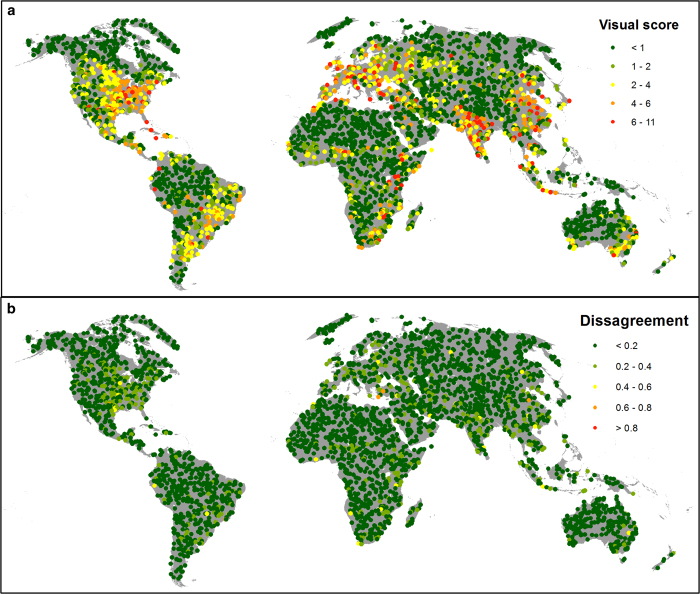
Results from 3114×1 km^2^ validation plots interpreted and scored following Supplementary Appendix 1. (**a**) The location and visually interpreted pressure score for plots, and (**b**) the disagreement between the Human Footprint score and the visual validation score on a normalized 0–1 scale.

High resolution images were used to visually interpret human pressures in 3460×1 km^2^ sample plots randomly located across the Earth’s non-Antarctic land areas (Fig. 3a). Images for these plots were obtained from World Imagery^52^, which provides one meter or better satellite and aerial imagery in many parts of the world and lower resolution satellite imagery worldwide. The map features 0.3 m resolution imagery across the continental United States and parts of Western Europe, as well as many parts of the world, with concentrations in South America, Eastern Europe, India, Japan, the Middle East and Northern Africa, Southern Africa, Australia, and New Zealand. The imagery used for the validation plots had a median resolution of 0.5 meters and a median acquisition year of 2010. Comparable imagery was not available for the 1993 time period, and therefore only the 2009 map underwent validation.

For the visual interpretation, the extent of built environments, crop lands, pasture lands, roads, human settlements, infrastructures and navigable waterways, were recorded using a standard key for identifying these features (Supplementary Appendix 1). Shape, size, texture and colour of features in the imagery were important characteristics for identifying human pressures on the environment. Interpretations were also marked as ‘certain’ or ‘uncertain’, and the year and resolution of the interpreted image was recorded. The 346 ‘uncertain’ plots were discarded, leaving 3114 validation plots (Fig. 3a). In general, plots were classified as ‘uncertain’ for two reasons; either because cloud cover obscured the image, or because only medium resolution (15 m) imagery was available for the plot, preventing accurate interpretation of the image. The human footprint score for each plot was determined in ArcGIS, and the visual and Human Footprint scores were then normalized to a 0–1 scale. As we only retained plots for which visual interpretations of the images were determined to be ‘certain’, we consider the visual score to be the true state of in-situ pressures for the plots.

Two statistics were used to determine Human Footprint performance, root mean squared error (RMSE)^53^ and the Cohen kappa statistic of agreement^54^. The RMSE is a dimensioned (expresses average error in the units of variable of interest) error metric for numerical predictions, and tends to heavily punish large errors. The Kappa statistic expresses the agreement between two categorical datasets corrected for the expected agreement, which is based on a random allocation given the relative class sizes. When calculating the kappa statistic, the Human Footprint score was considered as a match to the visual score if they were within 20% of one another on the 0–1 scale.

There is strong agreement between the Human Footprint measure of pressure and pressures scored by visual interpretation of high resolution imagery. The RMSE for the 3114 validation plots was 0.125 on the normalized 0–1 scale, indicating an average error of approximately 13%. The Kappa statistic was 0.737 (*P*<0.01), also indicating good agreement between the Human Footprint and the validation dataset. Of the 3114×1 km^2^ validation plots, the Human Footprint scored 94 of them 20% higher than the visual score and 263 of them 20% lower. The remaining 2757 plots (88.5%) were within 20% agreement. While this represents good agreement, it appears that the Human Footprint is to some extent susceptible to mapping pressures as absent in locations where they are actually present. The maps should therefore be considered as conservative estimates of human pressures on the environment. The Kappa statistic measure of agreement is sensitive to the threshold used to consider plots as a ‘match’. If we apply a more stringent threshold for agreement of within 15% of one another, the Kappa statistic falls to 0.565 (moderate agreement), and if we apply a less stringent threshold of within 25%, the Kappa statistic increases to 0.856 (very high agreement).

While agreement is generally strong, there is some geographic variation in the RMSE results comparing the Human Footprint scores and those derived from visual interpretation (Fig. 3b). By calculating RMSE for all biomes that contain at least 100 of the 3114 sample plots, we found that agreement was strongest in the Tundra biome and the Temperate grasslands, savannas and shrublands biomes (Table 4). Agreement was weakest in the Temperate broadleaf and mixed forest biome and the Boreal.

## Usage Notes

Mapping human pressures to the environment is an essential first step to identifying priority areas for protection or restoration of natural systems. Understanding the spatial distribution of pressures, as well as their change through time, also provides insights for studies on macro-ecological patterns. The Human Footprint maps for 1993 and 2009 represent the first temporally-consistent maps of the human footprint, as well as much more up-to-date information on cumulative pressures than is currently available. Moreover, the 2009 Human Footprint map is the first cumulative pressure map to have undergone an accuracy assessment.

The individual pressure maps were developed to be globally consistent, using a scoring approach originally developed by Sanderson and colleagues^13^. However, in some regions and for some species groups, alternate scores may be better suited for reflecting the pressures exerted by humans on nature. We therefore provide the individual pressure layers that compose the Human Footprint maps, thereby allowing data developers to create alternate scoring schemes that better suit their purposes, as well facilitating the addition of new or alternate data sources.

Moreover, our work is subject to three primary limitations. First, like all attempts to map cumulative pressures we did not fully account for all human pressures. Some of the missing and static pressures, such as invasive species and pollution, may be closely associated with pressures we did consider, and therefore their inclusion may not affect our overall results. Second, a lack of available data resulted in three of our pressures being static through time, which would cause an underestimation of Human Footprint expansion if these pressures expanded at a higher than average rate. Third, the Human Footprint measures the pressure humans place on nature, not the realized ‘state’ or ‘impacts’ on natural systems or their biodiversity. Significant scope exists to determine how natural systems respond to cumulating human pressures, and if non-linearity or thresholds exist where pressures lead to accelerated impacts.

While we welcome the opportunity to contribute intellectually and as co-authors to research projects that incorporate our datasets into their work, we make the data freely available without restriction for non-commercial use and redistribution. The data may be altered from their original form, and redistributed if done so free of charge and with a full description of any alterations to the original data. We do however ask that term ‘Human Footprint map’ be used only when referring to the unaltered data in the Human Footprint 7-zip file, and not to alternative versions of the data created by data users, and that the data be cited following the template at the end of this manuscript.

## Additional information

**How to cite this article:** Venter, O. *et al.* Global terrestrial Human Footprint maps for 1993 and 2009. *Sci. Data* 3:160067 doi: 10.1038/sdata.2016.67 (2016).

## Supplementary Material

Supplementary Appendix 1

## Figures and Tables

**Table 1:** Summary of data inputs, manipulations and outputs in the Human Footprint workflow

**Source**	**Data used**	**Temporal range**	**Resolution**	**Data manipulations**	**Outputs**
^REF. 27^	Average, stable lights, & cloud free coverages	1994, 2009	30 arc second, ~1 km at equator	1) Intercalibrate across years2) reproject and resample to 1 km raster basemap3) Convert to binary map of areas exhibiting a Digital Number equal to or above ‘20’.4) Assign these areas the pressure score of ‘10’.	Built1994.tifBuilt2009.tif
Data Citation 2	Gridded population of the world GPWv3, density grids	1990, 2010	2.5 arc minute, ~5 km at equator	1) reproject and resample to 1 km raster basemap2) Assign pressure score using equation (1) in methods	Popdensity1190.tifPopdensity2010.tif
^REF. 27^	Average, stable lights, & cloud free coverages	1994, 2009	30 arc second, ~1 km at equator	1) Intercalibrate across years2) reproject and resample to 1 km raster basemap3) Create 11 equal quintile bins for 1994.4) Assign pressure scores to bins from 0–10, for 1994, and using the same DN thresholds for 2009.	Lights1994.tifLights2009.tiff
Data Citation 3	University of Maryland Global Land Cover Classifications 1992–1993	1992–1993	1 km	1) reproject and resample to 1 km raster basemap2) Convert to binary map showing crop lands3) Exclude all areas already mapped as built4) Assign crop lands a pressure score of ‘7’.	Croplands1993.tif
^REF. 40^	GlobCover Version 2.3 2009	2005–2006	300 m	1) reproject and resample to 1 km raster basemap2) Convert to binary map showing crop lands3) Exclude all areas already mapped as built4) Assign crop lands a pressure score of ‘7’.	Croplands2005.tif
^REF. 41^	M3-Pasture data	2000	5 min, ~10 km at equator	1) reproject and resample to 1 km raster basemap2) Exclude all areas already mapped as built or crop lands3) Assign pressure score of 4, weighted by percent pasture lands	Pasturelands.tif
Data Citation 4	Global Roads Open Access Data Set (gROADS) v1	1980–2010	Vector data, accurate to 500 m	1) reproject and covert to 1 km raster basemap2) Exclude trails and private roads3) Assign pressure score of ‘8’ to roaded pixels, and ‘4’ to adjacent pixels, exponentially decaying to 0 at 15 km.	Roadways.tif
^REF. 30^	Vector Map Level 0 (VMap), railways	~1990	Vector data, accurate to 1 km	1) reproject and covert to 1 km raster basemap2) Assign pressure score of ‘8’ to rail pixels	Railways.tif
^REF. 48^	HydroSHEDS, stream discharge	No timeframe	3 arc second, ~100 m at equator	1) reproject and covert to 1 km raster basemap2) Use equations (2,3,4 to determine stream depth3) Exclude all stream reaches less than 2 m4) exclude all reaches no within 80 km of a stream bank which is within 4 km of a pixel with a DN>4, in 1994 or 2009.5) Add coastlines within 80 km of a coastal bank which is within 4 km of a pixel with a DN>4, in 1994 or 2009.6) Assign pressure score of ‘4’ to adjacent pixels, exponentially decaying to 0 at 15 km.	Navwater1994.tifNavwater2009.tif

**Table 2:** Pressure scheme used to assign weights to the eight individual pressures in the Human Footprint maps.

**Pressure**	**Score**	**Details**
Built environments	0,10	All areas mapped as build given score of 10.
Population density	0–10 Continuous	Pressure score=3.333×log (population density+1)
Night-time lights	0–10 Continuous	Equal quintile bins
Croplands	0,7	All areas mapped as crops given score of 7.
Pasture	0,4	All areas mapped as pasture given score of 4.
Roads	0,8 Direct impacts0–4 Indirect impacts	500 m either side of roads given a direct pressure score of 8Starting 500 m out from road, pressure score of 4 exponentially decaying out to 15 km.
Railways	0,8	500 m either side of railways given a direct pressure score of 8Starting 500 m out from road
Navigable waterways	0–4	pressure score of 4 exponentially decaying out to 15 km.

**Table 3:** The name, description and type of data included in the HumanFooptrint.zip file

**Name**	**Description**	**Format**
Validation.xlsx	Data on visual score of pressures for 3114 1 km^2^ plots using high resolution imagery.	Excel
Appendix1.pdf	Key used to visually interpret human pressures.	PDF
HFP1993.tif	The Human Footprint camp of cumulative pressures on the environment in 1993.	GeoTIFF
HFP2009.tif	The Human Footprint camp of cumulative pressures on the environment in 2009.	GeoTIFF
Built1994.tif	Individual pressure map of built environments in 1994.	GeoTIFF
Built2009.tif	Individual pressure map of built environments in 2009.	GeoTIFF
Croplands1992.tif	Individual pressure map of crop lands in 1992.	GeoTIFF
Croplands2005.tif	Individual pressure map of crop lands in 2005.	GeoTIFF
Lights1994.tif	Individual pressure map of night-time lights in 1994.	GeoTIFF
Lights2009.tif	Individual pressure map of night-time lights in 2009.	GeoTIFF
Navwater1994.tif	Individual pressure map of navigable waterways in 1994.	GeoTIFF
Navwater2009.tif	Individual pressure map of navigable waterways in 2009.	GeoTIFF
Pasture1993.tif	Individual pressure map of pasture lands in 1993.	GeoTIFF
Pasture2009.tif	Individual pressure map of pasture lands in 2009.	GeoTIFF
Popdensity1990.tif	Individual pressure map of human population density in 1990.	GeoTIFF
Popdensity2010.tif	Individual pressure map of human population density in 2010.	GeoTIFF
Railways.tif	Individual pressure map of railways circa 1990.	GeoTIFF
Roads.tif	Individual pressure map of roads circa 2000.	GeoTIFF

**Table 4:** Root Mean Square Errors results comparing the Human Footprint scores with 3114 validation plots globally, and for biomes with at least 100 plots within them.

Region	RMSE
RMSE Global	0.125706
RMSE Boreal	0.164053
RMSE Deserts and xeric shrublands	0.091757
RMSE Montane grasslands	0.121541
RMSE Temperate broadleaf and mixed forests	0.175661
RMSE Temperate grasslands, savannas, and shrublands	0.085226
RMSE Tropical and subtropical grasslands, savannas, and shrublands	0.121362
RMSE Tropical and subtropical moist broadleaf forests	0.142398
RMSE Tundra	0.028995
