# Supplementary material for: Global terrestrial Human Footprint maps for 1993 and 2009
Source: Sci Data. 2016 Aug 23;3:160067. doi: 10.1038/sdata.2016.67 (PMC5127486; doi:10.1038/sdata.2016.67)
Supplement: Supplementary Appendix 1 [file sdata201667-s1.pdf]

Appendix 1. Visual interpretation of satellite images for mapping human pressures

When interpreting images, interpreters can zoom in and pan to identify pressures. For sample areas where only coarse scale Landsat images are available, these images can be used if it is deemed that they are sufficient to allow classification for the area, which may be possible in highly green wilderness areas. Otherwise, the sample should be marked as ‘na’. If the shape matches expectations, cleared patches with bare ground are assigned the land cover category of the wider landscape, eg. urban, forestry or crops. This is done as bare ground across a plot within farm land is likely to be tilled farmland, likewise a brown patch in a forested landscape is likely to be a recently felled clear cut. Distinguishing between crops and pasture is a challenge, zooming in to look for linear planting lines or signs of cattle or their feeding/drinking points may help. Some land cover types are not mutually exclusive, for instance, urban areas may also be scored as high density for roads and human dwellings. Crops, pasture, urban and forestry are mutually exclusive at a site, but can co-occur within a $1km^2$ or $100km^2$ sample area. Following visual interpretation, interpreters should mark their interpretation as ‘certain’ or ‘not certain’. Certain means that 95% of the time you will be right. The year of images for all is accessed for all samples using the information tool and recorded.

The samples are selected using a random sampling. Those are automatically overlaid with ESRI high resolution images within ArcGIS 10.1, allowing a rapid access to recent remote sensing images with zooming capabilities. For a given sample the expert saw not only the sample point but also a box that coincided with the so-called observational unit and its quadrats.

Figure 1 The level of detail of images available in many locations. In the first panel, horses can be seen grazing in front of the farm house, and hay bales can be seen wrapped and stacked to the right of the barn. In the second panel, the uniform grey of concrete, as well as individual containers and the cranes used to move them can be seen. Shape, size, texture and colour are important characteristics for identifying human pressures on the environment.

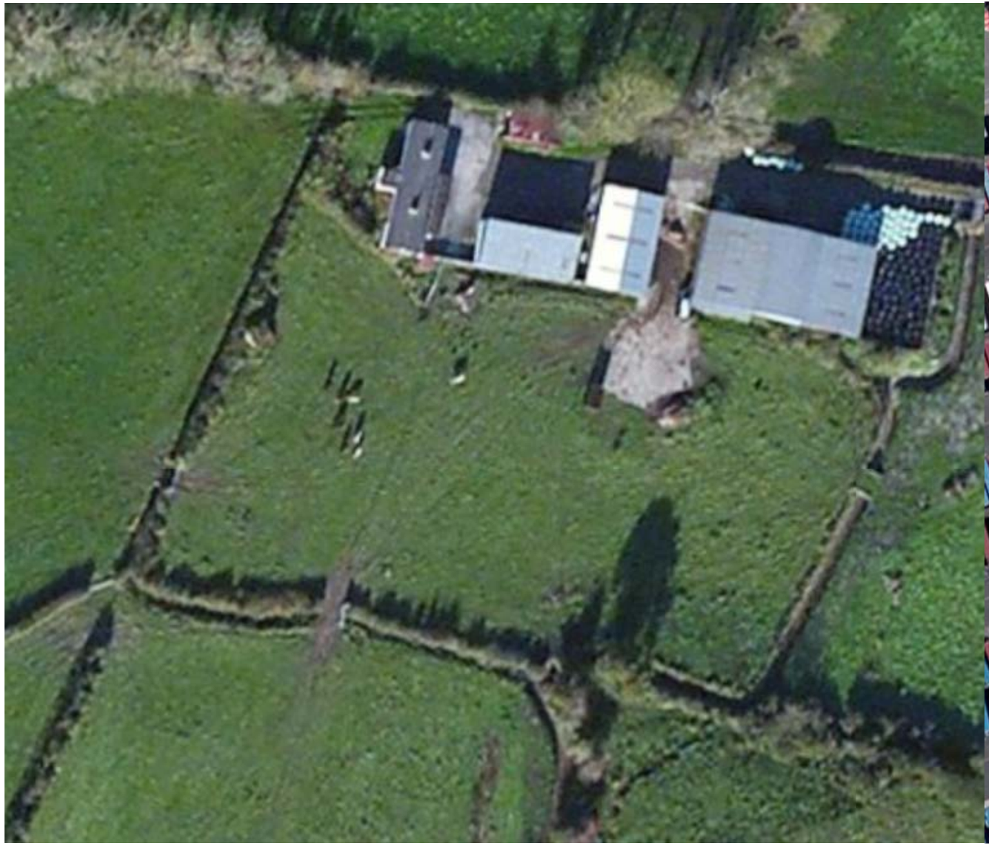

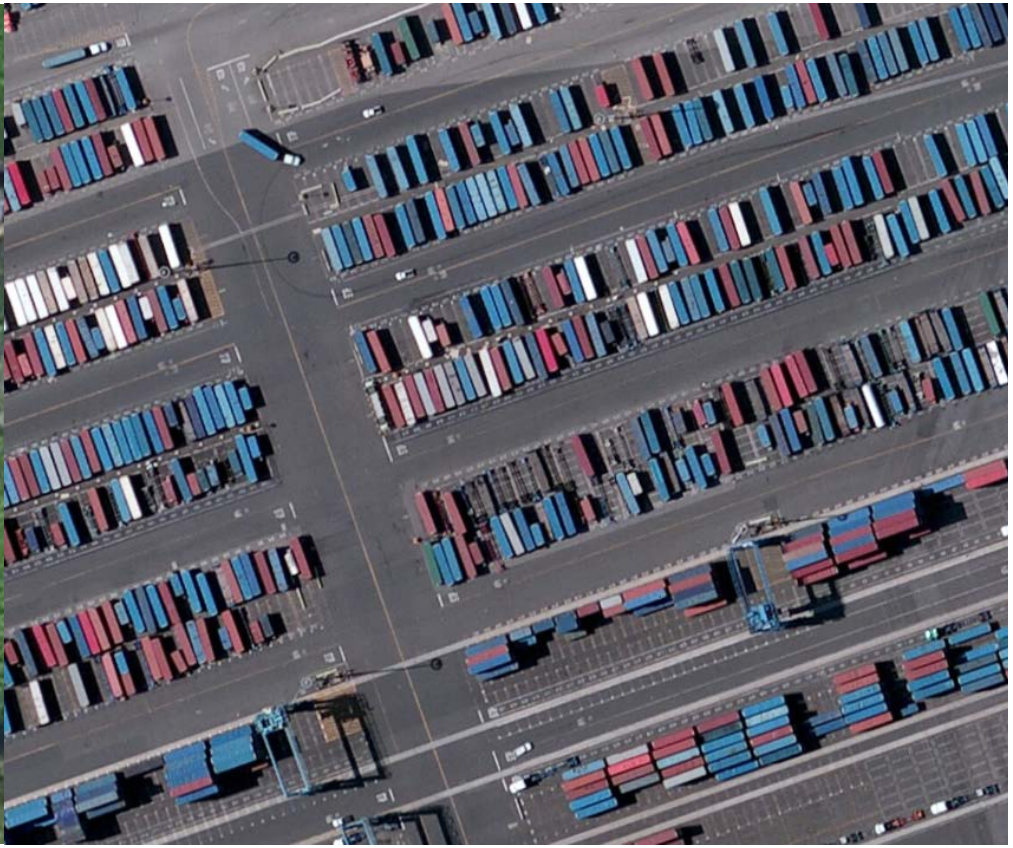

Pressure	Description	Scoring
Urban	Built environments are human produced areas that provide the setting for human activity. These are primarily urban settings, including buildings, paved land and urban parks, and excludes isolated roads and isolated housing. They are easily identified by sharp contrasts in tones, widespread homogeneous grey surfaces, and recognisable human constructed shapes. % built? Definition of urban. Urban park is a highly managed natural vegetation.	None = 0,<br>sparse = 1, <12.5%<br>medium = 2, >12.5%<br>dense = 3, >50%

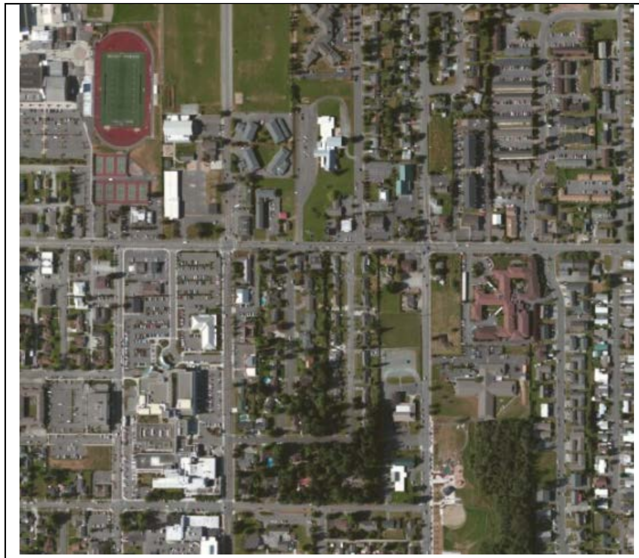	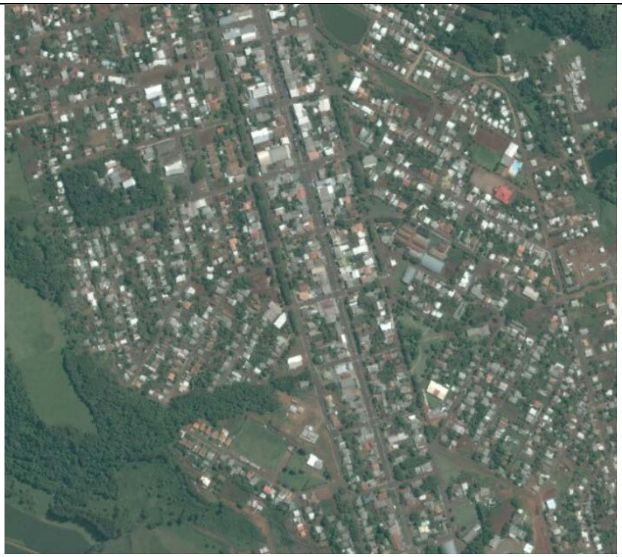	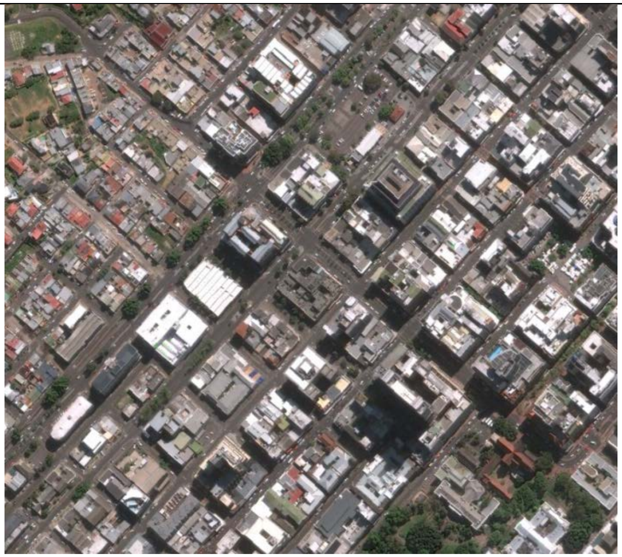
Crops	Croplands are cultivated areas used for annual or perennial crops, such as orchards or vineyard. Typically exhibit a checkerboard pattern of cropland pattern from different crop stages (exhibited by varying grey tones) and differences in tillage directions. Cropland areas, generally devoid of trees, possess a smoother texture than pasture land areas and often have linear markings from planting, harvesting or tilling lines.	None = 0,<br>sparse = 1, <12.5%<br>medium = 2, >12.5%<br>dense = 3, >50%
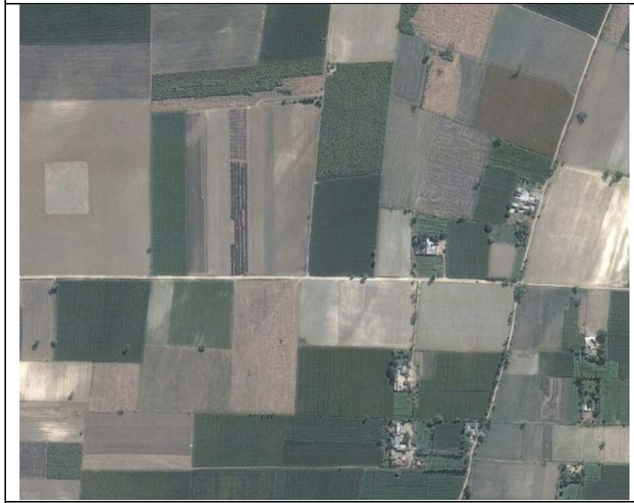	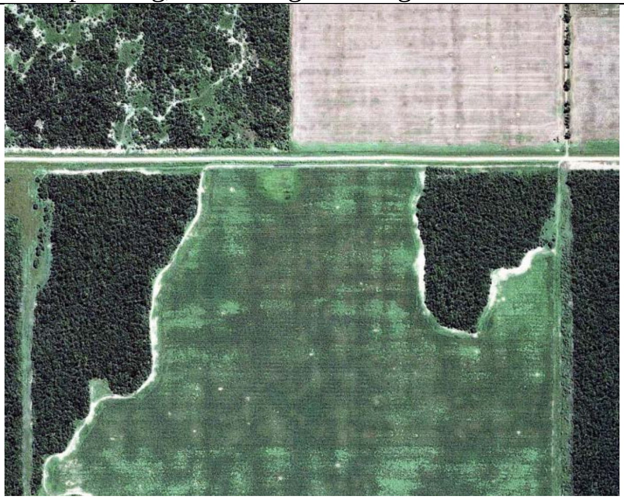	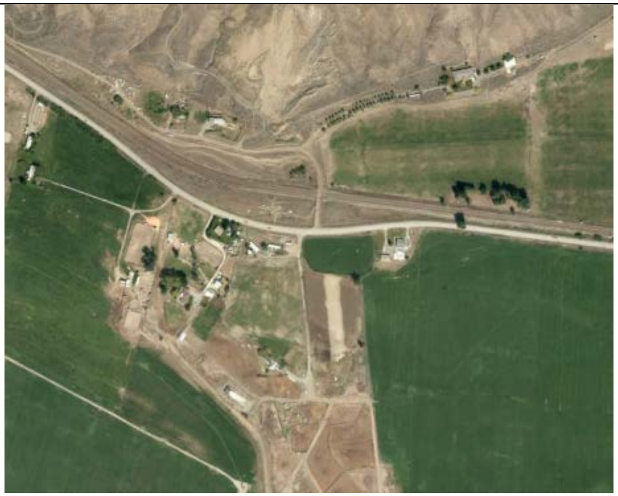
Pasture	Land covered with grass and other low plants suitable for	None = 0,

	grazing animals, especially cattle or sheep. Often characterized by fencing without linear cropping, but often with linear changes in vegetation blocks along fence lines. Cattle or their tracks, as well as vehicle access tracks may be visible.	sparse = 1, <12.5%<br>medium = 2, >12.5%<br>dense = 3, >50%
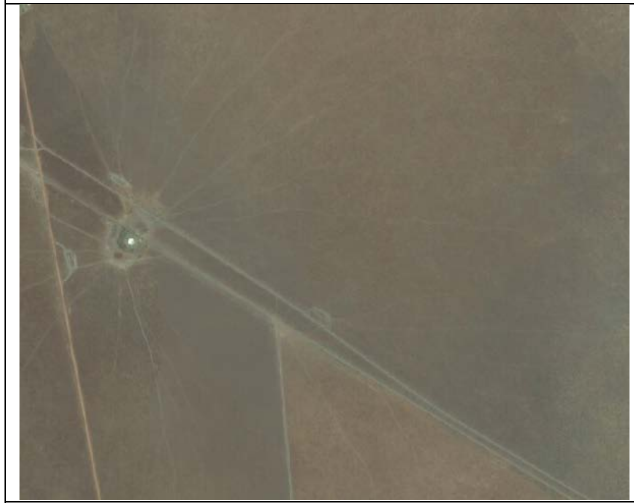	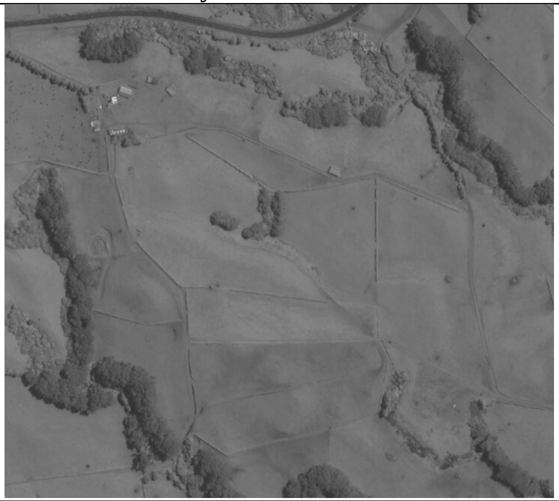	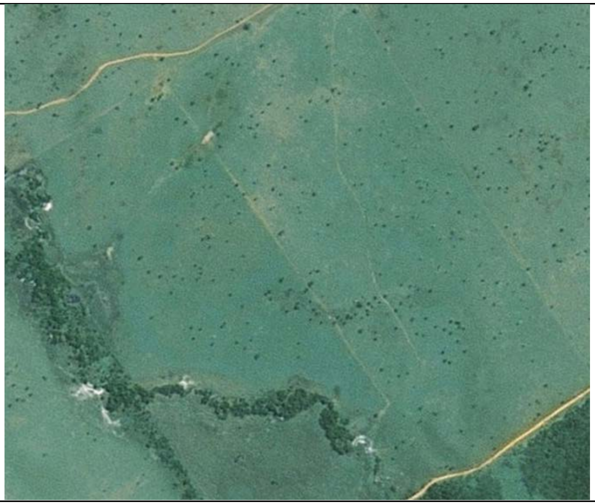
Roads-paved, unpaved and private	Linear infrastructure with a wide homogeneous grey surface, and often a disturbed vegetation or bare earth band in parallel. Paved roads have a grey surface, unpaved roads have a brown surface. Private roads are not used for transportation by the public, but rather provide private access, such as access to farm fields.	None = 0,<br>sparse = 1, at least one road visible<br>medium = 2, roads with length that traverses the image twice<br>dense = 3, roads with length that traverses the image 5 times

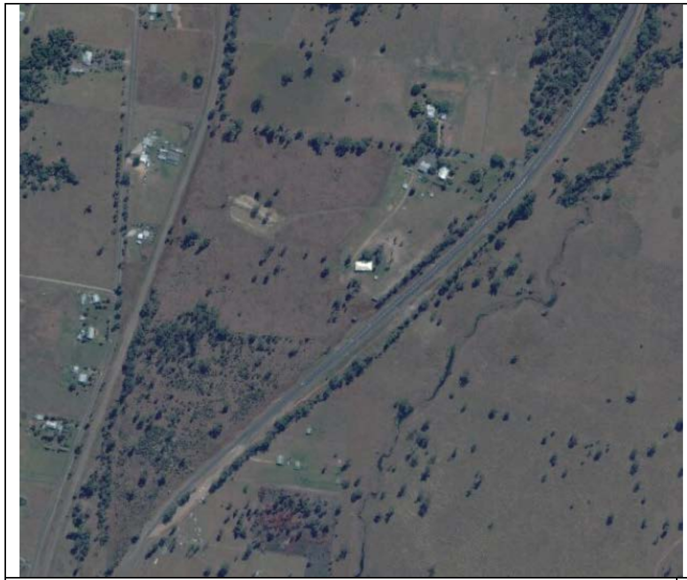	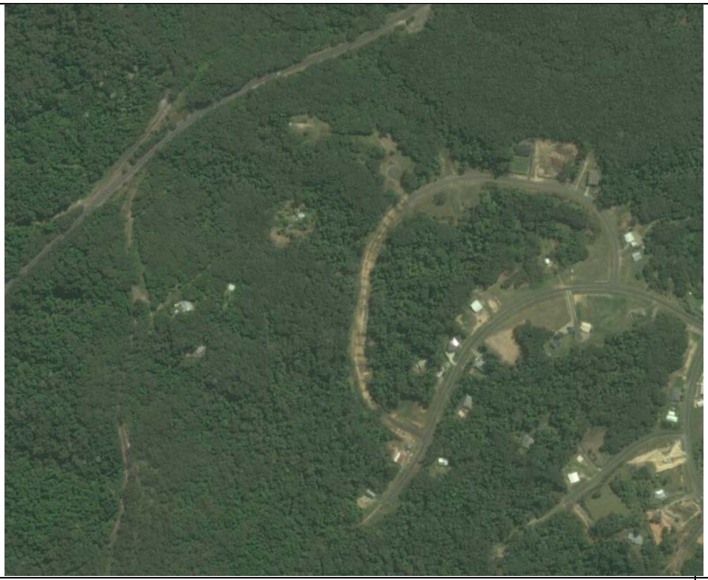	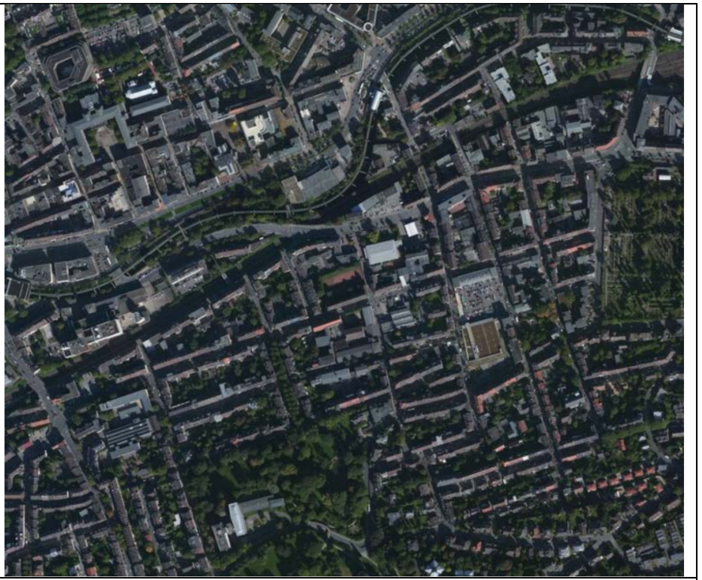
Forestry	Harvesting of natural or plantation forest. Can be clear-fell harvesting, common in temperate forests, or selective logging, common in the tropics. Clear-fell harvesting characterized by large patches of felled forest of often irregular shape following topographic features. Selective harvesting characterized by much smaller harvest patches, a network of dirt roads with noticeable small cleared areas with dirt surface used for landing logging. Selective logging common in the tropics. Plantation forests can be distinguished by their uniform tree cover, and sometimes linear planting rows.	None = 0,<br>sparse = 1, <12.5%<br>medium = 2, >12.5%<br>dense = 3, >50%

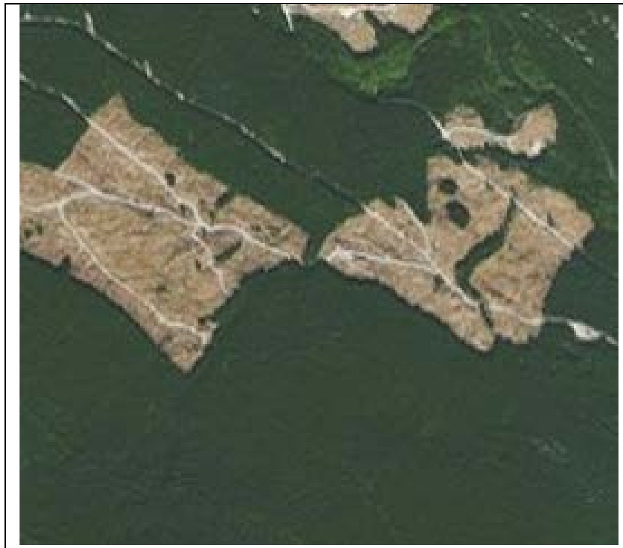<br>Clear cut logging in British Columbia, Canada	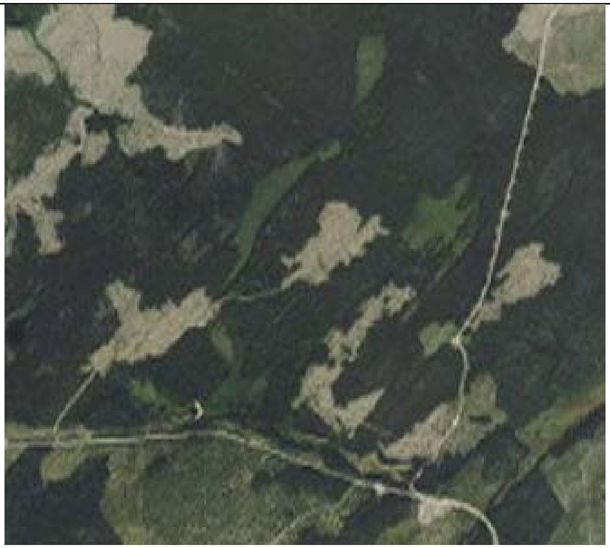<br>Clear cut logging in Russia.	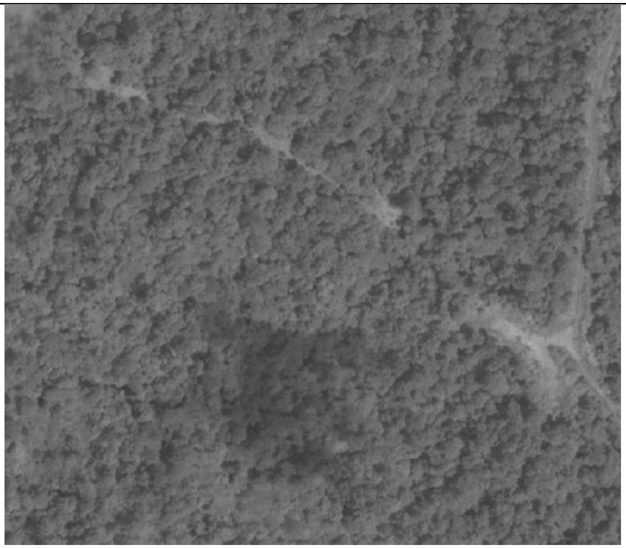<br>Selective logging in Indonesian Borneo
Human dwellings	Human dwellings, including dense urban areas with apartment buildings, and sparser suburban and rural housing	None = 0,<br>sparse = 1, <4 single-family dwellings per km^2<br>medium = 2, <20 single-family dwellings per km^2<br>dense = 3, >20 dwellings per km^2, or 1 apartment building per km^2
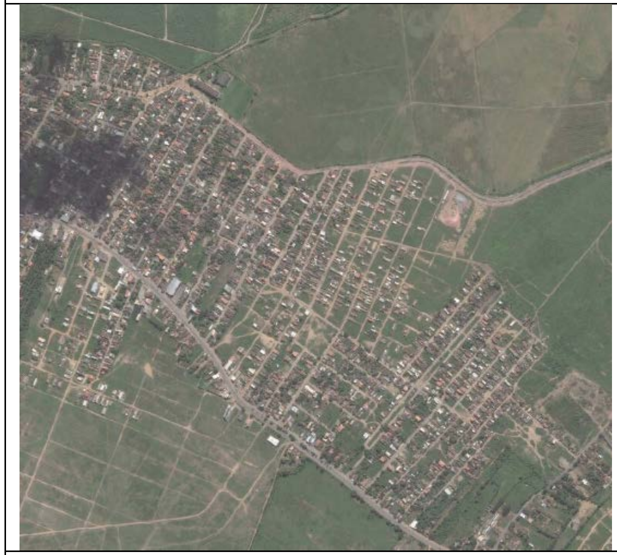	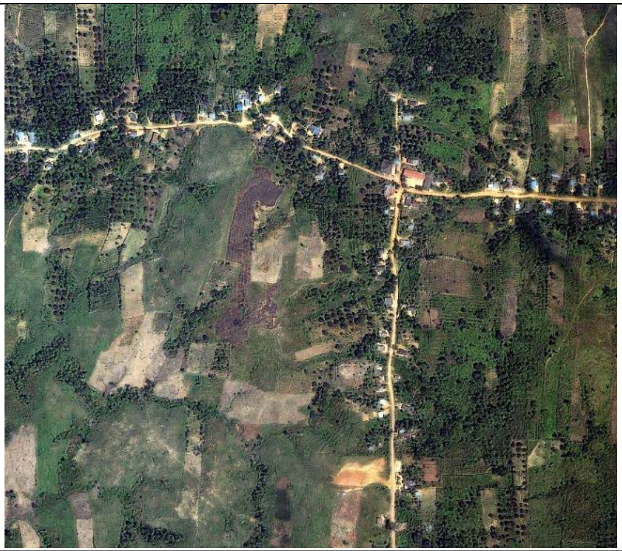	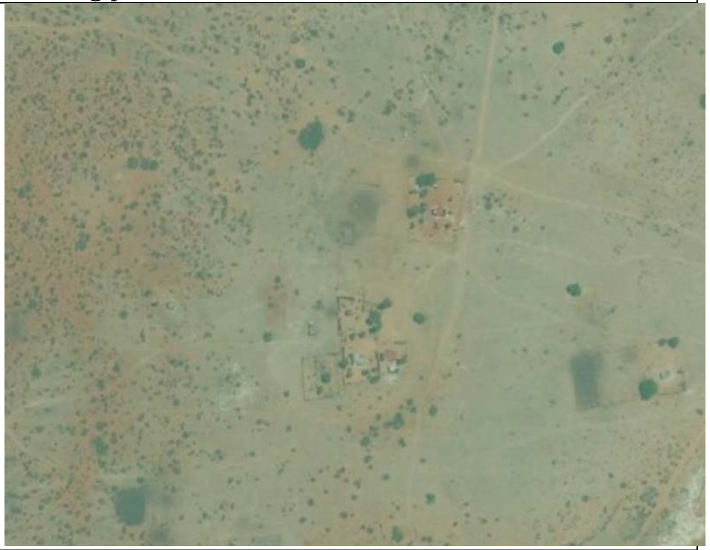
Infrastructure	Non-urban, housing, road or rural infrastructure. Includes Mining infrastructure, dam infrastructure, rail, and other	None = 0,<br>sparse = 1, <12.5%

	linear features such as pipelines.	medium = 2, >12.5%<br>dense = 3, >50%<br><br>and for linear infrastructure:<br><br>None = 0,<br>sparse = 1, at least one road visible<br>medium = 2, roads with length that traverses the image twice<br>dense = 3, roads with length that traverses the image 5 times
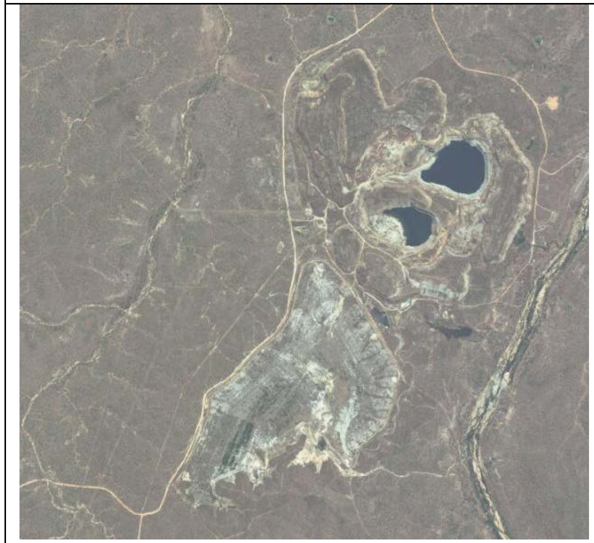<br>Mining site sub-Saharan Africa	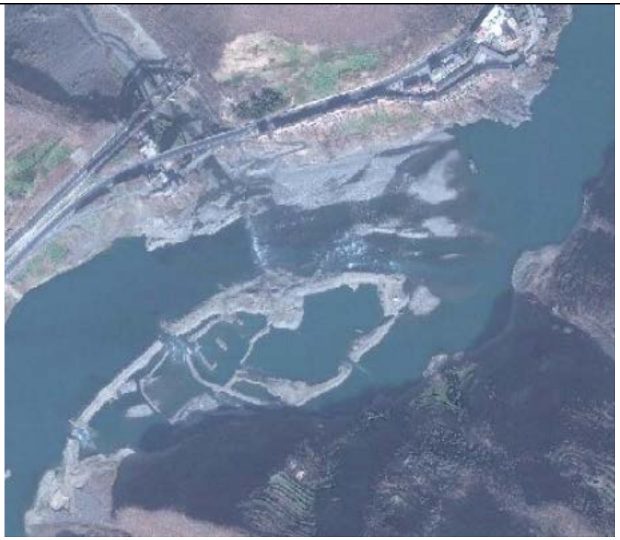<br>Industrial and dam infrastructure in China	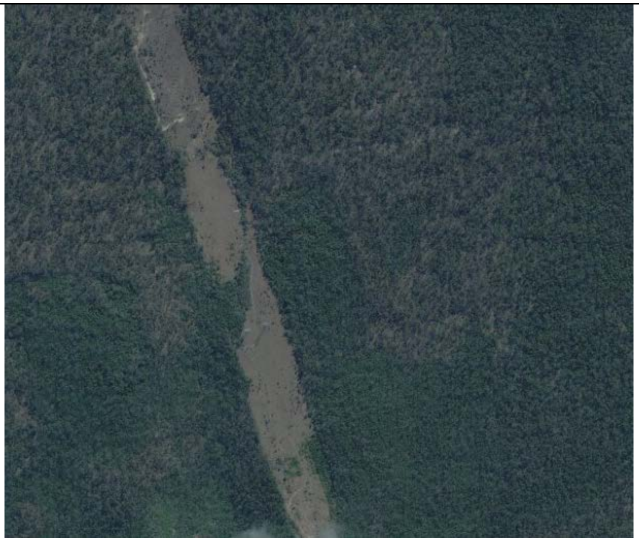<br>Electricity transmission line in Australia
Navigable waterways	Navigable waterways appear wide and deep enough for a vessel to travel, and lack impassable areas of whitewater. Signs of human activity along the shoreline, such as human structures or roads leading to the water within 40km of the sample plot mean the waterway is likely to be navigated.	None = 0,<br>sparse = 1, at least one navigable waterway<br>medium = 2, navigable waterways with length that traverses the image twice<br>dense = 3, navigable waterways with length that traverses the image 5 times
